# Hyperlocal lessons from the COVID-19 pandemic: Toward an equity-centered implementation science approach

**DOI:** 10.1016/j.ssaho.2024.100844

**Published:** 2024

**Authors:** Brian J. Manns, Stephen Thomas, Oluyemi Farinu, Makhabele Woolfork, Chastity L. Walker

**Affiliations:** a Centers for Disease Control and Prevention, Atlanta, GA, USA; b Maryland Center for Health Equity, University of Maryland, College Park, MD, USA

**Keywords:** Implementation science, Health equity, COVID-19, Community-based participatory research, Hyperlocal approaches

## Abstract

COVID-19 vaccination campaigns across the US were implemented to mitigate the disproportionate hospitalizations and unnecessary deaths across many communities that experienced unequal gaps in initial vaccine distribution rollout and uptake. In parallel, the COVID-19 pandemic created declines in routine vaccination coverage for adults, adolescents, and children; particularly, in communities experiencing overlapping social disadvantage. Community-based efforts offer a solution to narrow immunization gaps but have not been replicated consistently nor demonstrated widespread success during the pandemic as evidenced by prevailing disparities in immunization uptake. We offer an equity centered implementation science approach that involves co-designing, co-implementing, and co-evaluating solutions with the community and all partners investing in the shared goal of sustainable improvement in health outcomes.

## 1. The COVID-19 pandemic and vaccination gaps in the U.S.

Vaccinations are important for protecting communities from preventable illnesses that can cause life-threatening health complications. Anderson (Anderson, 2014) ^(p344)^ describes vaccines as, “the foremost achievement of public health programs in the United States.” Still, the initiation of large-scale COVID-19 vaccination campaigns across the country unintentionally resulted in unequal gaps in vaccine access and uptake across many communities. In parallel, the COVID-19 pandemic created declines in routine vaccination coverage for adults, adolescents, and children; particularly, in communities experiencing overlapping social disadvantage (Hill et al., 2023; Hong et al., 2021; Seither et al., 2023). Missed opportunities for vaccination can stem from circumstances beyond one’s control (Dinleyici et al., 2021). It is here where non-medical factors beyond the individual generate population health inequities across a myriad of social determinant of health domains. For example, limited access to reliable transportation options, economic hardship, and concentrated poverty contribute as root causes of disparities associated with inequitable vaccination uptake and poor health outcomes (Viswanath et al., 2021; Williams et al., 2022).

There are many hurdles to vaccine confidence and uptake which can generally be categorized into three buckets: *structural, behavioral, and informational*. Structural barriers refer to physical or logistical obstacles that prevent access to immunization services. These types of barriers may include limited employee benefits like paid time off for lower income workers. Some literature suggest structural barriers reflect differential access to societal resources and disproportionately impact racially and ethnically minoritized communities, owing to the fundamental effects of structural racism and its role exacerbating disparities in adult vaccination coverage (Fisk, 2021; Njoku et al., 2021). Behavioral barriers can refer to individual or cultural factors that influence vaccination decisions, such as uncertainty of new and rapidly developed vaccines or prior unpleasant interactions with the healthcare system (Chu & Liu, 2021). Informational barriers refer to lack of awareness or understanding about the importance of vaccination, misinformation and how to access services. Very early into the pandemic, when there was heightened concern for exposure to the virus causing COVID-19, many jurisdictions issued orders intending to curb community-level transmission. While well intentioned these actions resulted in people delaying and avoiding preventive care (Czeisler, 2020). In turn, this created fewer logistical opportunities for receiving recommended immunizations; thus, exacerbating structural barriers (Santoli, 2020).

The pandemic has led to greater proliferation of misinformation about vaccines, driving behavioral and informational barriers (Barua et al., 2020). Together, these barriers reinforce the social determinants of health that limit accessible vaccination opportunities and one’s ability to reach optimal health (Peña et al., 2023; Brakefield et al.). As a result, these communities are at increased risk not only for COVID-19 disease, but other vaccine preventable diseases that produce life-threatening health outcomes (Abrams & Szefler, 2020). Efforts that minimize barriers and protect persons in socially disadvantaged communities should be prioritized and further explored.

## 2. All public health is local

Hyperlocal solutions employ the integration of localized health strategies and interventions in well-defined community/geographic locations thereby prioritizing the needs of populations and leveraging existing community-based infrastructure and systems supporting well-being. To rectify disparities, programs intending to eliminate gaps in routine immunization coverage should consider the full range of community needs in collaboration with other sectors that play a role in addressing the barriers described above (Thomas et al., 2011). But what guides how public health practically does this? In 2015, the Centers for Disease Control and Prevention (CDC) Community Preventive Services Task Force attempted to review and synthesize evidence supporting community-based programs that increase vaccination rates in targeted populations (Guide to Community Preventive Service, 2015). This review highlighted the effectiveness of community-based programs in low socioeconomic groups in addition to racially and ethnically minoritized populations. The report concluded community-based interventions are shown to increase vaccination rates in targeted populations when implemented as two or more coordinated interventions. The extent to which evidence-based coordinated vaccination programs have been replicated in communities across geographic and population settings, or how coordinated vaccination programs in community settings contributed to narrowing immunization uptake disparities in the U.S. throughout the pandemic is currently unknown as efforts have not been rigorously evaluated.

Due to limited evidence, there are, to date, no best practices guiding optimal vaccination program design, implementation, and approaches to evaluation of coordinated community vaccination programs to produce the intended outcomes that advance equity (Loper et al., 2021). However, researchers are beginning to document the role of conceptual frameworks and constructs for community engagement and partnerships which may support increased equitable vaccination coverage. Early evaluation approaches of these equity centered operational considerations highlight the importance of addressing local structural factors affecting communities can result in improved vaccination opportunities in underserved community settings (Assoumou et al., 2022). Therefore, centering equity in the implementation of community vaccination programs hold promise. Commitment to equity-centered implementation science involves co-designing, co-implementing, and co-evaluating solutions with the community and all partners investing in the shared goal of sustainable improvements in health outcomes (Brownson et al., 2021). It centers community priorities, local values and expertise for public health programs looking to successfully reduce unjust and avoidable differences in health. The application of equity-centered implementation science also empowers communities to take part in the decision-making process. For interventions to meet the specific needs of the community, we should consider their priorities to overcome barriers and achieve intended health outcomes.

Despite a diverse range of activities targeting disparities, there is a heightened awareness for social determinants of health influences exacerbating predictable health inequalities during a future pandemic threat should public health not heed lessons learned and prepare appropriately (Hoven et al., 2022). Public health practitioners are at a moment to reflect on interventions impacting traditionally underserved populations and accelerate their dissemination to advance health equity. Many coalitions have been built, new approaches tested, and practices deemed promising (Linnan et al., 2022). The extraordinary deployment of innovative interventions requires ongoing support and maintenance to learn how these efforts further protect and uplift the hardest-hit and highest-risk communities. This will require a systematic approach that elevates the voices of communities in a way that produces health impact. Demonstration of the continuing commitment to equitably elevate and prioritize diverse perspectives during the COVID-19 pandemic response was reflected in the White House Summit on COVID-19 Equity and What Works Showcase (The White House, 2022) co-hosted by the US Centers for Disease Control and Prevention on November 16, 2022. The Summit highlighted various interventions and approaches across populations that have moved the needle on equitable COVID-19 outcomes. The goal of the Summit was to convene community equity leaders, government, and philanthropy to showcase a path forward for equitable vaccination program implementation in local settings. Gaps in COVID-19 multidose primary series vaccination coverage rates between Black, Latin-o/Hispanic adults and white adults were nearly closed (Kriss, 2022), due in part to hyper local approaches prioritizing disproportionately impacted populations. The table below offers examples of hyperlocal community driven approaches used to mitigate inequities in routine immunization services. (See Table 1). Many organizations and individuals have contributed to this historic proof of concept for how long-standing inequities can be overcome at a large scale driven by coordinated vaccination programs at the community-level.

In addition to empowering underrepresented voices and legitimizing authentic experience as knowledge, equity-centered implementation science also allows additional exploration for generalizability of findings and external validity in health services research (Brownson et al., 2022). A typical challenge with public health interventions is understanding the context behind why an intervention may or may not be effective when translating success across populations and settings. Critical dissemination of effective hyper local practice-based community vaccination models in real-world settings is a necessary step toward equitably improving health outcomes.

## 3. A need for equity-centered implementation science

Equity-centered implementation science is important because it offers an approach that addresses both historical and contemporary dynamics contributing to disparities and mistrust in government across various socially disadvantaged communities. Historical events such as the U.S. Public Health Service Syphilis Study at Tuskegee and government forced sterilizations are regularly referenced as contributors to long standing mistrust of the medical system among racial and ethnic minority communities (Gamble, 1997; Volscho, 2010; Washington, 2006). However, contemporary health issues such as maternal mortality facing Black, Hispanic, and American Indian communities further demonstrate how structural racism at the systems level is one domain of a multi-factorial pathway generating inequitable health outcomes (Howell, 2018). Other forms of disadvantage that disrupt health include ableism, medical racism, and gentrification, the equally harmful economic action of disinvestment in communities (Feagin & Bennefield, 2014; Fuentes et al., 2021; Schnake-Mahl et al., 2020). Advancing health equity necessitates the public health field dismantle the compounding systems of disadvantage that negatively impact communities. Initiatives like the National Institute of Health – Community Engagement Alliance and CDC’s Partnering for Vaccine Equity represent practical public health examples toward equity in vaccination access, confidence, and uptake. Both activities assembled hundreds of organizations and trusted community members working alongside state and local health departments to empower community voices, build toward stronger collaborative partnerships, and operate an infrastructure that could narrow long standing vaccination inequities (Koppaka et al., 2023; Mensah & Johnson, 2024). Rather than imposing poorly designed and ineffective solutions, the experiential knowledge of communities from the federal COVID-19 vaccination programs described and others can guide coordinated vaccination public health efforts. Learning to fully lean on the expertise of communities, may allow for greater success in addressing health disparities.

Putting theory to practice in alleviating disparities and gaps in routine immunization requires a focus on addressing the social determinants of health. This includes finding solutions for underlying social and economic conditions that contribute to inequitable differences in health status. Centering equity in hyperlocal approaches to address gaps in vaccination can be achieved through several tactics, including:

1. **Uplifting Community Leaders as Researchers and Health Extenders**: Recognizing and valuing the expertise of community members ensures the diverse perspectives, needs, and wants are centered in research efforts. This can be done by providing training and support that strengthens community member’s skills in recognizing population health challenges, implementing appropriate health intervention strategies, conducting research, and participating in research narratives largely dominated by academics unfamiliar to local contexts. Supporting a strong cadre of local equity leaders as investigators and healthcare extenders who bring community knowledge, values, and experience may offer opportunities to further extend public health interventions and intervention adaptations deeper into communities.
2. **Community-Based Participatory Research (CBPR)**: CBPR is a research approach that involves equal partnership between researchers and community members. It allows for the community to be involved in every aspect of the research process, from identifying research questions to analyzing data and disseminating results. By engaging in CBPR, scientists can ensure that their research is relevant, actionable, and responsive to community needs. Furthermore, this allows for trust building, bi-directional learning, and community ownership.
3. **Adequate and Sustained Funding for Implementation Science**: Advancing health equity among persons who are historically underserved by a variety of systems requires significant investment. Underfunding of any program intended to improve health damages trust with communities and offers no sustainability. Support for community engagement, education, and community-based participatory research further champions the first strategy for uplifting community researchers. Because the factors contributing to poor health are varied, a layered approach that involves actions across multiple levels (eg., individual, community, physical, social, and political) is necessary to produce and reinforce change. In order to better understand the drivers at the individual and community levels that make a public health program successful and that also advance equity by demonstrating gains in health, funding for implementation science is needed. Sustainable funding also demonstrates a commitment to change and contributes to improved outcomes.
4. **Identifying Community Assets**: The strengths, resources, and capacities that exist within communities can aid in the co-design of efforts and programs. In doing so, this can help to ensure interventions meet the needs of communities and promote longevity. Engaging with communities to establish shared goals and expectations reinforces accountability. Moreover, transparent communication amongst all stakeholders upholds fairness and mutual responsibility in the decision-making process.
5. **Keep the Equity Focus Going**: Starting with equity in mind, front and center, is critical. Addressing inequities and gaps in health outcomes is a long-term process that requires sustained effort. Recognizing that addressing issues related to race and other social determinants is not a sprint but a marathon.

## 4. Implications and benefits for future study

A literature review synthesizing over 20 years of published adult racial and ethnic vaccination disparity research underscored the lack of progress in identifying and implementing actionable strategies impacting the upstream social determinants of health affecting vaccination uptake (Granade et al., 2022). The large focus on individual level factors impacting vaccination uptake has allowed the public health field to understand the power of trusted messengers and provider recommendations in moving individuals along the continuum toward vaccination acceptance. However, current research does not demonstrate optimal approaches to overcoming upstream social determinants of health or explain components of community vaccination programs which spur engagement and improved equitable vaccination uptake. On the ground knowledge and experiences from communities can offer perspective toward the development of impactful vaccination programs in the future while building an infrastructure for strong community and local health department partnership structures. Public health practitioners can evaluate hyperlocal community factors that support impactful vaccination program development and implementation, particularly among those at high risk of experiencing serious health complications or death.

The COVID-19 pandemic created a heightened awareness of longstanding health inequities. Hyperlocal approaches are a crucial component to any program intending to alleviate vaccination disparities, build trust with communities and ensure all communities have equitable access to routine vaccinations. By implementing these approaches and maintaining an equity focus, improved overall health outcomes across populations becomes obtainable. Public health in collaboration with other sectors have a responsibility to confront the complex web of structural factors that contribute to inequitable immunization coverage and uptake. Advancing health equity is challenged by time, resources, and societal will. Failure to support these strategies can diminish gains made in hyperlocal public health capacity building and further perpetuate longstanding disparities across communities. Implementation science, centered around equity, promotes optimal public health practice, shares power with communities, and can improve vaccination program interventions.

## Figures and Tables

**Table 1:** Examples of effective hyper local practice-based efforts.

Hyper Local Principle	Community Approach	Barriers and Challenges Addressed (Structural, Behavioral, Informational)	Outcomes (Unpublished Data shared with CDC from community organizations)
Partner public health agencies and health delivery systems with community-based organizations with reach to socially disadvantaged groups	Local health department hired and partnered with members of refugee, immigrant, and migrant communities to create culturally and linguistically appropriate education materials and outreach plans. Trusted and convenient community locations offering additional opportunities for clinical care and referral to social services were identified.	Structural barriers (e.g., access to quality care provided in traditional settings) Behavioral barriers (e.g., mistrust in institutions and prior negative experiences with health systems)	Hired three Community Health Navigators from local communities; Administered 304 COVID-19 vaccinations; Distributed 1180 home test kits and 900 masks
Engage community leaders and members as trusted messengers to promote vaccination, address vaccine hesitancy, and bolster confidence	American Indian tribal leaders and elders organized as trusted messengers and culture keepers bridging connections to land, language, and health with COVID-19 vaccination, and reinforcing support for immunization.	Informational barriers (e.g., limited internet and cell phone accessibility, linguistic barriers)	Engaged 83,000 people through educational events and 5000 individuals via other platforms-tablet, text, and phone; 1 million media/social media impressions (radio, ads, and op-eds);
Pursue diverse representation and inclusion through the provision of translation services and culturally tailored education	A US Territory based independent living center addressed the needs of people with diverse disabilities (e.g., created accessible educational materials, enlisted sign language interpreters, hosted vaccination events in communities or at homes of bedridden persons)	Informational barriers (e.g., limited access to interpreters, cultural and language communication barriers) Structural Barriers (e.g., lack of transportation to and from vaccination sites and limited vaccine access in low-income areas) Behavioral barriers (e.g., negative experiences when providers do not know how to interact with persons with disabilities)	Improved accessibility and vaccine coverage for persons with disabilities; Hosted 38 accessible vaccination events hosted; Administered 832 COVID-19 vaccinations
Develop integrated solutions that remove multiple barriers to accessing vaccination and care	A community health center worked with local community-based organizations to introduce a 34-foot mobile medical unit, providing bundled services-vaccination, primary care, and dental, to persons experiencing homelessness, persons with substance use disorders, and those who may engage in sex work.	Structural barriers (e.g., access and compounding lived experiences) Behavioral barriers (e.g., mistrust of government and health care systems)	Administered 30,000 COVID-19 vaccines; Conducted 35,000 COVID-19 vaccine education sessions; Engaged 32 vaccine ambassadors (18 with lived experience); Created new partnerships across sectors (e. g., fire services, law enforcement, harm reduction organizations, food programs, libraries, and recreational centers
